# Regular three-dimensional presentations improve in the identification of surgical liver anatomy – a randomized study

**DOI:** 10.1186/1472-6920-13-131

**Published:** 2013-09-25

**Authors:** Beat P Müller-Stich, Nicole Löb, Diana Wald, Thomas Bruckner, Hans-Peter Meinzer, Martina Kadmon, Markus W Büchler, Lars Fischer

**Affiliations:** 1 Department of Surgery, University of Heidelberg, INF 110, Heidelberg, 69120, Germany; 2 Division of Medical and Biological Informatics, German Cancer Research Center (DKFZ), INF 305, Heidelberg 69120, Germany; 3 Institute for Medical Biometry and Informatics, Heidelberg 69120, Germany

## Abstract

**Background:**

Three-dimensional (3D) presentations enhance the understanding of complex anatomical structures. However, it has been shown that two dimensional (2D) “key views” of anatomical structures may suffice in order to improve spatial understanding. The impact of real 3D images (3Dr) visible only with 3D glasses has not been examined yet. Contrary to 3Dr, regular 3D images apply techniques such as shadows and different grades of transparency to create the impression of 3D.

This randomized study aimed to define the impact of both the addition of key views to CT images (2D+) and the use of 3Dr on the identification of liver anatomy in comparison with regular 3D presentations (3D).

**Methods:**

A computer-based teaching module (TM) was used. Medical students were randomized to three groups (2D+ or 3Dr or 3D) and asked to answer 11 anatomical questions and 4 evaluative questions. Both 3D groups had animated models of the human liver available to them which could be moved in all directions.

**Results:**

156 medical students (57.7% female) participated in this randomized trial. Students exposed to 3Dr and 3D performed significantly better than those exposed to 2D+ (p < 0.01, ANOVA). There were no significant differences between 3D and 3Dr and no significant gender differences (p > 0.1, t-test). Students randomized to 3D and 3Dr not only had significantly better results, but they also were significantly faster in answering the 11 anatomical questions when compared to students randomized to 2D+ (p < 0.03, ANOVA). Whether or not “key views” were used had no significant impact on the number of correct answers (p > 0.3, t-test).

**Conclusion:**

This randomized trial confirms that regular 3D visualization improve the identification of liver anatomy.

## Background

Because of its proven effect on spatial understanding, three-dimensional (3D) representations are increasingly used not only in the clinical setting but also in the education of both students and patients [1-7]. The reasons for the better spatial appreciation are well known and include better recognition of “key views” [8,9]. In addition, the computer-based conversion from 2D to 3D allows for optimization of the presented information [10,11]. The clinical routine, however, is almost exclusively characterized by 2D images (e.g., CT, MRI).

One goal of the research at our department is dedicated to the question of how to improve the identification of surgical liver anatomy [2,3,12-15]. As stated above, 3D presentations are considered to be superior in the identification of complex 3D structures. It has been shown, however, that providing students with multiple two-dimensional “key views” of anatomical structures can also improve the understanding of spatial anatomy [8]. These intriguing differences were the basis of the first question to be answered by this randomized study—does providing students randomized to 2D with four additional “key views” (2D+) improve their ability to identify liver anatomy?

If one looks at the studies describing 3D, the appropriate images are not truly three-dimensional. That is, these so-called 3D images do not use the third dimension but rather apply techniques such as shadows and different grades of transparency to create the impression of 3D. A real perception of depth is missing. We were able to establish a real 3D (3Dr) effect by using anaglyph images of the human liver. These images were used to determine the effect of 3Dr images on the identification of liver anatomy, which was the second goal of this randomized study.

## Methods

**Figure 1:**
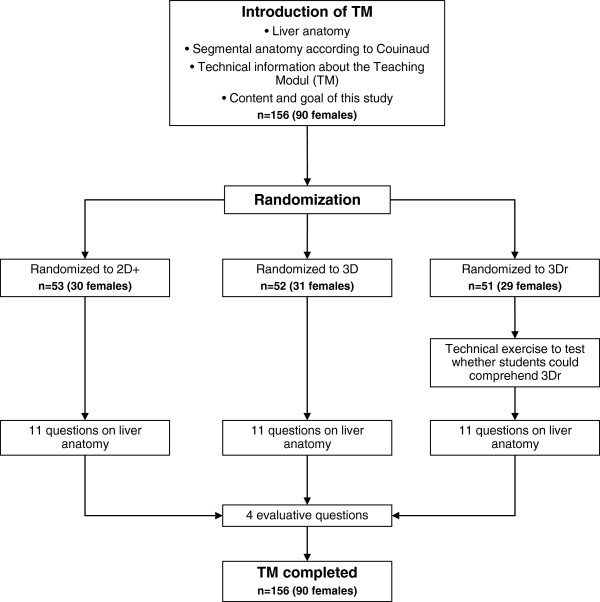
Flow chart of the study design.

A computer-based teaching module (TM) was used based on the open source toolkit Medical Imaging Interaction Toolkit (MITK) [16,17]. During this study, the TM was tested on medical students at a certain stage in their graduate training (third or fourth year). All participants had completed their basic training in anatomy, learning anatomy not only through books and dissecting corpses, but also with the help of computer simulations. Before starting the TM, a lecture was given to the students. This PowerPoint© presentation described the liver anatomy and the definition of Couinaud liver segments in both 2D and 3D. In addition, a short technical description of the TM was given to the students (see Figure 1). To start the TM, each student had to enter gender, year of graduate training, and a unique randomization code (letter randomization). Each random group was given only one modality—either 2D+, 3D or 3Dr—and was tested within the randomized modality. No time frame was given to finish the TM.

**Figure 2:**
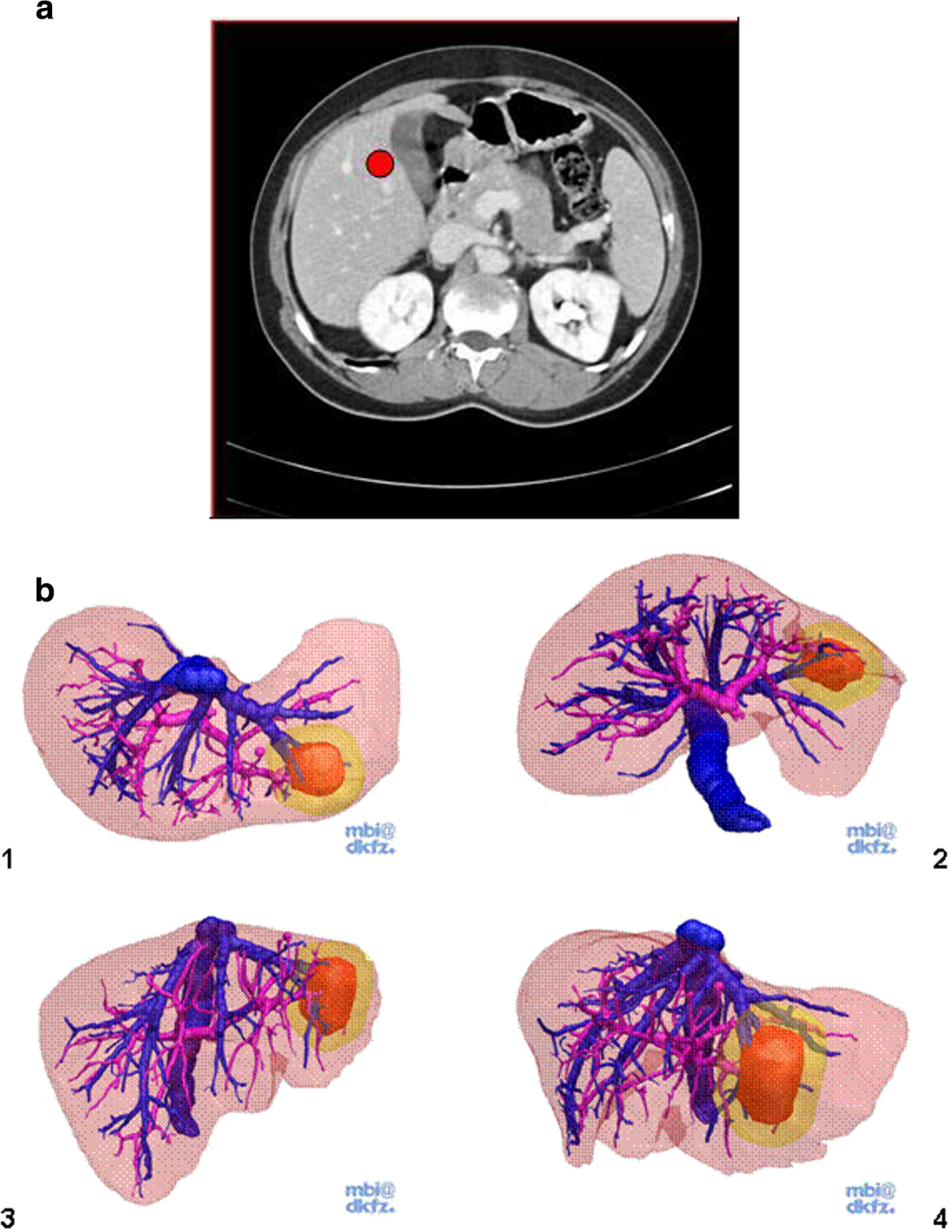
**a Screenshot showing the TM for 2D+.** A regular CT movie was presented as consecutive transverse slices. Students could freely scroll up and down. The red circle represents the main task of question #1 in which students were asked to define in which CS this particular mark was set. **b** Together with the CT movie, students were provided with four “key views” of the same liver with views from above (1) and below (2) as well as a frontal image (3) and a left lateral view (4). The tumor in CS 2 and 3 was the basis for questions #7–#11.

**Figure 3:**
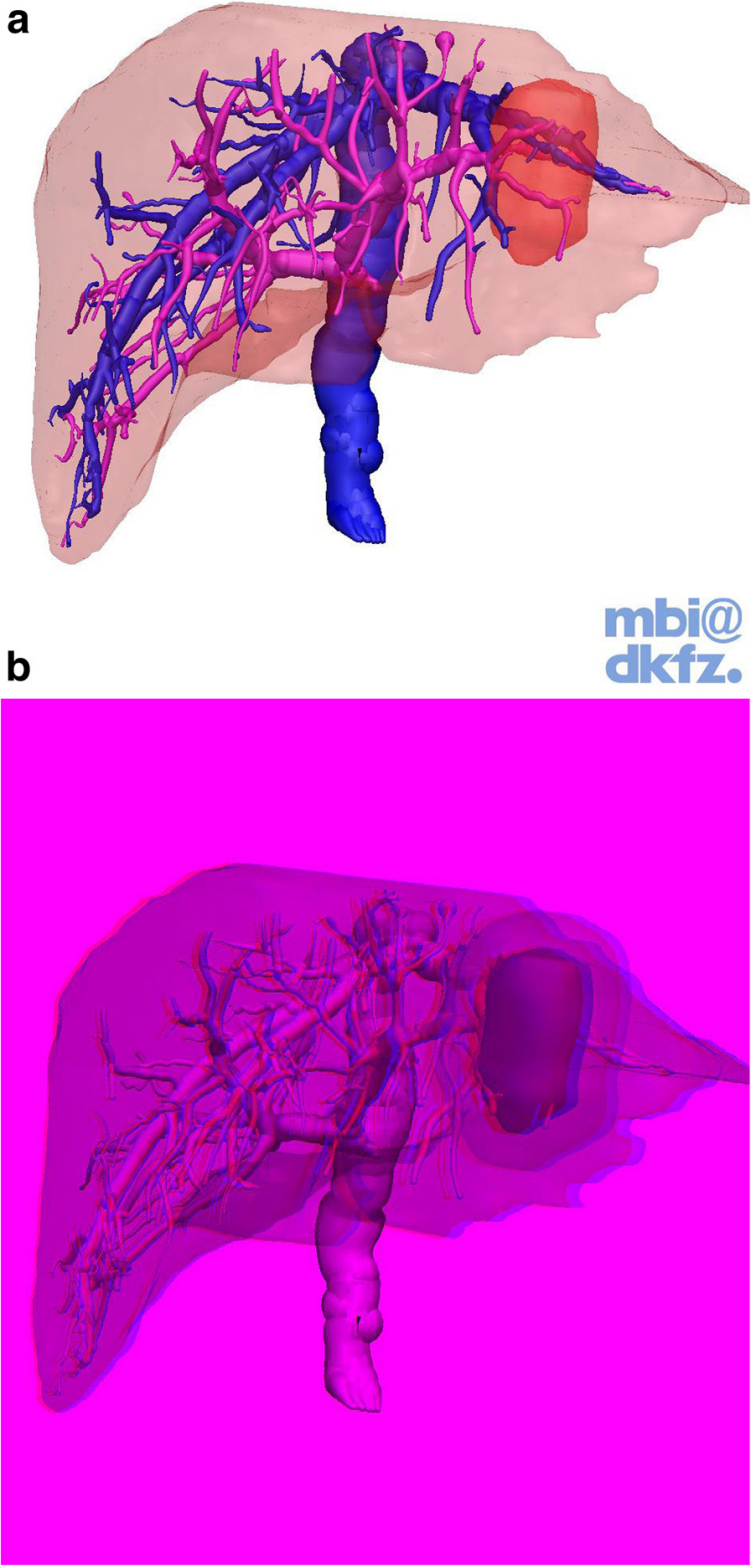
**a Screenshot showing the TM for 3D.** The cylindrical shaped tumor in CS 2 and 3 was the basis for questions #7–#11. **b** Screenshot showing the TM for 3Dr. This Figure can only be appreciated with 3D glasses (red and cyan glasses). The cylindrical shaped tumor in CS 2 and 3 was the basis for questions #7–#11.

Images of a contrast-enhanced liver CT in a portal venous phase (Figure 2a) presented as consecutive transverse slices plus four “key views” of the same liver (2D+; Figure 2b), regular 3D presentations (3D; Figure 3a) and “real” three-dimensional presentations only visible with 3D glasses (red and cyan glasses, 3Dr, Figure 3b) were available to help answer 11 questions concerning surgical liver anatomy (Table 1). The alignment of the 4 key views presented to the students were labeled accordingly as “view from above” (1), “view from below” (2) as well as a “frontal view” (3) and a “view from left lateral” (4). After finishing the 11 questions, all students were asked to answer the 4 evaluative questions (Table 1).

**Table 1:** Setting, question, and choice of answers for the 15 questions

**#**	**Setting**	**Question**	**Answers**
1.	A mark was placed in Couinaud segment (CS) 5.	In which Couinaud segment is this mark placed?	One possible answer from CS 1 - 8 (Figure 1)
2.	The software of the TM is able to differentiate between the vessel tree of middle, right, and left hepatic veins.	Please click on any branch of the middle hepatic vein!	The placed dot was automatically recognized by the TM as correct or incorrect.
3.	A branch of the portal vein is highlighted.	Which vessel is highlighted?	1 = hepatic vein
			2 = hepatic artery
			3 = portal vein (correct)
			4 = bile duct
4.	A branch of the hepatic vein is highlighted.	Which vessel is highlighted?	1 = hepatic vein (correct)
			2 = hepatic artery
			3 = portal vein
			4 = bile duct
5.	The software of the TM is able to differentiate between all 8 CS.	Please click on any vessel branch in CS 7!	The placed dot was automatically recognized by the TM as correct or incorrect.
6.	A mark was placed in the right liver half.	Which liver half is this mark placed in?	1 = left hemi liver (correct)
			2 = right hemi liver
7.	An artificial tumor was placed in CS 2 and 3.	Which CS is this tumor located in?	Possible answers from CS 1 to CS 8
8.	An artificial tumor was placed in CS 2 and 3.	Which vessels run through the tumor?	1 = left hepatic vein (correct)
			2 = right hepatic vein
			3 = left portal vein (correct)
			4 = right portal vein
9.	An artificial tumor was placed in CS 2 and 3.	Please describe the shape of the tumor.	1 = rotund
			2 = cone-shaped
			3 = cylindrical (correct)
			4 = egg-shaped
10.	An artificial tumor was placed in CS 2 and 3.	How many CS have to be resected in order to remove the tumor completely?	1 = 1 CS
			2 = 2 CS (correct)
			3 = 3 CS
			4 = 4 CS
11.	An artificial tumor was placed in CS 2 and 3.	Please guess which percentage of the liver must be resected to remove the tumor completely!	Students are asked to enter a number ranging from 0% to 100%.
12.	No imaging modality is provided to the participants.	Did you have fun performing this teaching module?	Possible answer: rating from 1 (not at all) to 5 (very much)
13.	No imaging modality is provided to the participants.	Do you think that the existence of the other imaging modality would have been helpful?	1 = yes
			2 = maybe
			3 = no
14.	No imaging modality is provided to the participants.	How would you rate your personal learning effect?	1 = high
			2 = regular
			3 = low
15.	No imaging modality is provided to the participants.	Would you feel confident presenting and explaining the Couinaud liver segmental anatomy to your fellow students?	1 = yes
			2 = yes, with some uncertainty
			3 = yes, but with significant uncertainty
			4 = no

After answering the 15 questions in total (11 medical and 4 evaluative questions), the 2D+ group had one additional question in which they were asked to state which of the presented four “key views” were helpful for them (students could choose any combination of the four “key views” including “all” and “none”). The decision on the four “key views” was made by our group, which consisted of one radiologist, one computer specialist, one surgeon and two medical students. In our opinion, these four key views would enable medical students to identify liver anatomy much better and help them to answer more questions correctly. The number of four key views is a combination of what we thought to be important views and the fact that these four key views are easily presented on one regular sheet of paper.

Before starting with the 11 medical and 4 evaluative questions, students randomized to 3Dr had to perform a rather technical exercise. To assess whether they could see/understand the 3Dr effect, students had to answer whether object A was in front of or behind another object B. This was repeated three times in different combinations.

Both 3D presentations (3D, 3Dr) could be turned interactively in all three dimensions, and students could zoom, pan and tilt the images.

All results (correct/not correct) and the time needed to answer each question were automatically recorded. Answers were only considered correct or incorrect; there was no partial credit for partially correct answers (for instance, question #10). In case of question #11, we allowed for a range of ±15% to consider the answer correct.

The distribution of continuous data was described with mean, standard deviation and minimum, median and maximum. In the case of categorical data with absolute and relative frequencies (count and percent), possible differences between groups were evaluated using an analysis of variance in the case of continuous data and a chi-square test in the case of categorical data. In case of overall significant differences as a result of ANOVA, pair-wise t-tests where evaluated using the closed testing procedure. Box plots were used to visualize the distribution of continuous data. The level of significance was set to 5%. All statistical calculations were carried out using SAS Version 9.1 WIN (SAS inc. Cary NC, USA).

Ethical approval was obtained by the Ethikkommission der Medizinischen Fakultät Heidelberg (S-082/2010).

## Results

A total of 156 graduate students (57.7% female) participated in this randomized trial, all participants being in their third or fourth year of training. The gender distribution was similar between the three groups (Table 2).

**Table 2:** Number of participants and gender distribution as randomized to any of the three imaging modalities (2D+, 3D, and 3Dr)

**Modus**	**n (%)**	**Female students (%)**
2D+	53 (33.97%)	30 (56.60%)
3D	52 (33.33%)	31 (59.61%)
3Dr	51 (32.69%)	29 (56.86%)
Total	156 (100%)	90 (57.69%)

**Figure 4:**
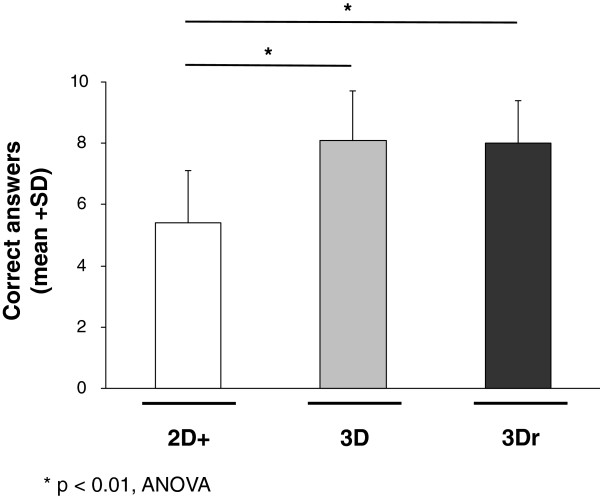
Sumscore of correct answers achieved in each image modality (2D+, 3D and 3Dr).

**Figure 5:**
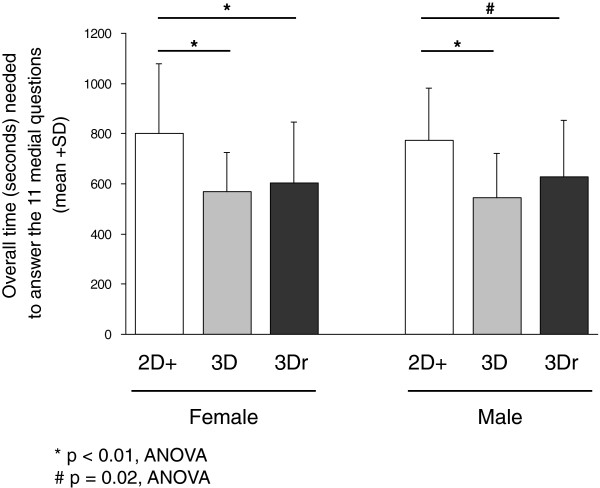
Overall time (seconds) needed to finish the 11 questions of the TM.

Overall, students exposed to 3D (mean sumscore 8.1, SD 1.6) and to 3Dr (mean sumscore 8.1, SD 1.4) performed significantly better than those exposed to 2D+ (mean sumscore 5.4, SD 1.7; Figure 4; p < 0.01, ANOVA). There were no significant differences between 3D and 3Dr (Figure 4; p > 0.1, t-test). Looking at the individual questions, there were no significant differences in the number of correct answers between 2D+, 3D, and 3Dr concerning questions #5, #10, and #11 (p > 0.3, chi-square test). For all other questions, however, students randomized to either 3D or 3Dr had significantly more correct answers compared to students randomized to 2D+ (p < 0.04, chi-square test). There were no significant gender differences within the three randomized groups (p > 0.1, ANOVA; data not shown). Students randomized to 3D and 3Dr had not only significantly better results, but they were also significantly faster in answering the 11 anatomical questions than students randomized to 2D+ (p < 0.03, ANOVA, Figure 5).

**Figure 6:**
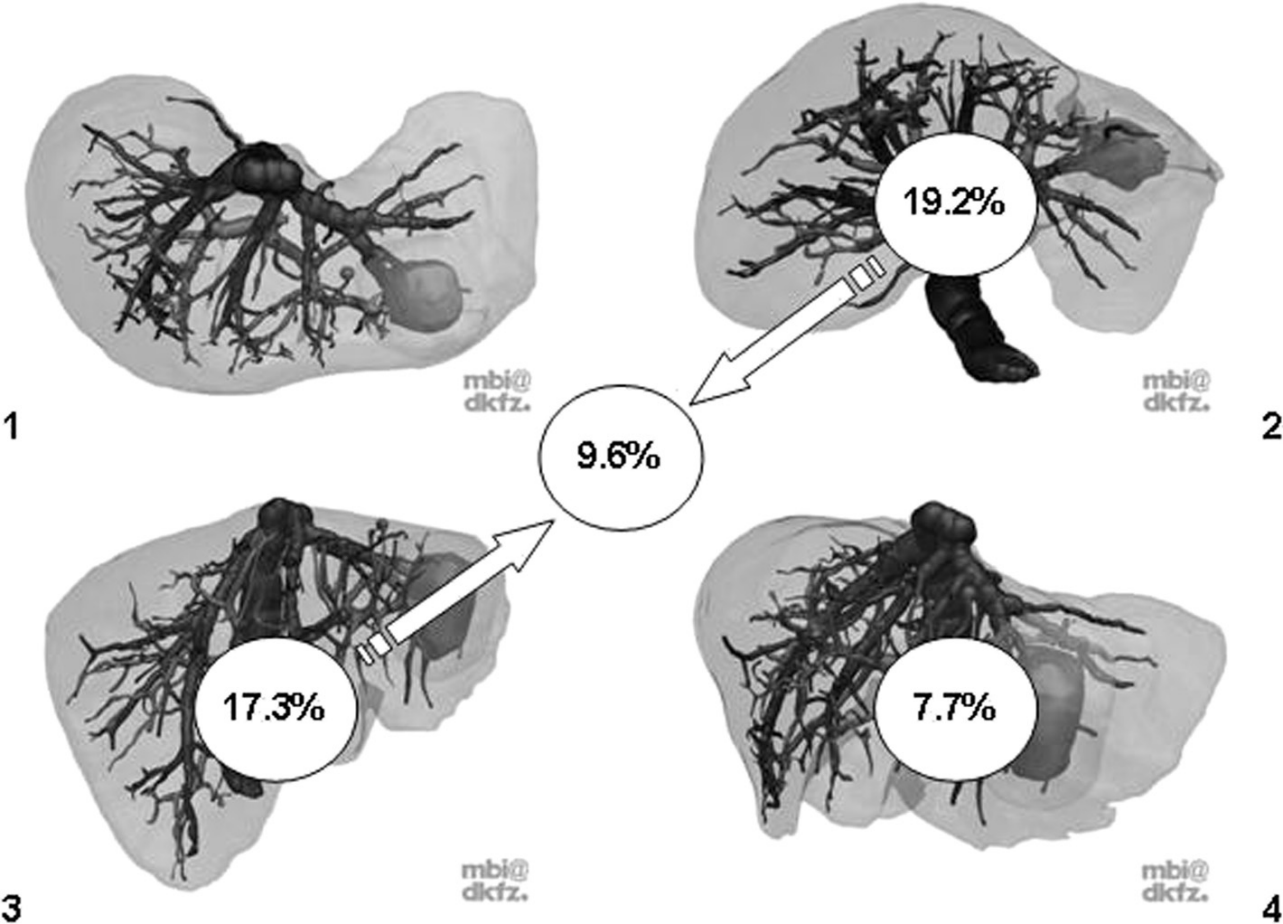
**Frequency (%) of how often the “key views” (alone or in any combination) were considered helpful by students randomized to 2D+.** The tumor in CS 2 and 3 was the basis for questions #7–#11.

As stated in the Material and Methods section, students randomized to 2D + had four additional “key views” of the same liver available; 9.6% of these students stated that all images were useful. In contrast, 23.1% of students found none of the provided images helpful. In analyzing which images were of particular help, image 2 (19.2% of students), image 3 (17.3% of students), the combination of both images (9.6% of students) and image 4 (7.7% of students) proved to be helpful (Figure 6). All other images or image combinations were selected by less than 5% of the students. However, whether or not the image/s were considered helpful by the students had no significant impact on the number of correct answers (p > 0.3; ANOVA, data not shown).

Overall, participating students enjoyed this TM. However, students randomized to 3D (94.2%) and 3Dr (90.2%) rated “fun” (question #12) significantly more often with “very much” or “much” when compared to 2D+ (56.6%; p < 0.01, chi-square test; data not shown). When asked whether the other imaging modality would be helpful (question #13), students randomized to 2D+ answered significantly more often with “yes” than those randomized to 3D or 3Dr (41% (2D+) vs. 21% (3D) vs. 17% (3Dr), p < 0.01, chi-square test; data not shown). The learning effect (question #14) was rated with “high” or “very high” significantly more often by students randomized to 3D (86.8%) and 3D + (76.5%) compared to 2D+ (50.9%; p < 0.01, chi-square test; data not shown). Subsequently, significantly more participants randomized to 3D or 3Dr felt that they could present their knowledge about the CS (question #15) without or with minor uncertainty to other students (2D+: 60.9%, 3D: 84.6%, 3Dr: 70.6%; p = 0.01, chi-square test; data not shown).

## Discussion

The benefits of three-dimensional learning tools are no longer doubted. Nowadays, there are well-powered randomized studies available supporting this statement [3,18-20]. However, under clinical conditions, radiological images are most often presented in 2D (e.g., CT, MRI). Based on the fact that “key views” of the appropriate anatomical structure can improve understanding of spatial anatomy [8], the first goal of this randomized study was to examine whether the additional presentation of key images with the regular 2D CT images could enhance the identification of liver anatomy.

So far, articles dealing with three-dimensional visualization have not actually used the third dimension. Instead, they have relied on a “pseudo” 3D effect, which is based on shadows and different grades of transparency. In the data presented here, “real” 3D images were used which could only be properly examined while looking through special 3D glasses. The second goal of this randomized study, thus, was to determine the effect of “real” 3D images on liver anatomy identification.

The results of the 156 students from this study allow three main conclusions. Firstly, the addition of multiple “key views” presented to students randomized to 2D did not have a significant effect on correct answers. Secondly, the presentation of real 3D images did not further improve the identification of surgical liver anatomy. Thirdly, students randomized to 3D and 3Dr not only show significantly higher sumscores when compared to students randomized to 2D, but they were also significantly faster in answering the 11 medical questions.

The results observed in this study on 156 students were rather unexpected. We do not have a clear explanation as to why the addition of four “key views” did not result in improved sumscores. The students randomized to 2D+ were told that the four “key views” represented the same liver as shown in the CT scans. However, the students apparently did not find the “key views” particularly supportive since the biggest proportion of students (23.1%) found none of the provided images helpful. Most likely the “key views” were not optimally chosen even though our interdisciplinary group (including two medical students) put considerable effort in the selection. Selecting fixed “key views” simply may not meet the individual needs of each person. This goes along with findings of other authors [8] where it has been shown that students, given the choice, use many different perspectives in order to understand anatomy. One way to avoid this scenario in the future would be either to offer multiple “key views” or to allow students to pick their personal “key views” in advance. Another explanation for the missing effect of the “+” in 2D+ may be that the students were exclusively occupied with trying to understand the CT images, and thus were not able to include the given information of the additional “key views” in their decision making.

The same would be true for the missing effect of 3Dr. Looking at 3Dr images with 3D glasses was impressive; however, this effect did not result in significant increases in the sumscores. Here, some students complained that their eyes hurt after a while and they had to remove the glasses. There were 4 out of 51 students (7.8%) who had at least one mistake in the 3Dr pre-test, which was designed in determine whether students could really comprehend 3Dr (see Material and Methods section). However, there were no significant differences in the sumscores between students who had no mistakes in the pre-test compared to students with at least one mistake. One could argue that the use of anaglyph stereoscopic images might be suboptimal since there are better formats for producing stereo images. However, the software for anaglyph stereoscopic images was already in use. Further, the computers and their graphic software were 5–6 years old and we were not sure if they could handle more advanced formats.

Reflecting on the results of all 156 students, it was evident that both 3D groups consistently rated the level of fun significantly higher than the 2D groups. The game character of the 3D animation might easily explain this since all 3D models were freely movable. This therefore makes a strong argument for developing teaching tools that students enjoy—having “fun” may help students to comprehend things faster. However, “fun” has to be considered as “add on” issue; it cannot replace the impact of a time-consuming, intensive engagement with the studied topic.

## Conclusion

This randomized trial shows that regular 3D visualization improves the identification of liver anatomy. Additions such as real 3D or key views have no significant impact.

## Abbreviations

CS: Couinaud segment; TM: Teaching module; 2D+: Two-dimensional CT images presented together with four “key views”; 3D: Three-dimensional presentations; 3Dr: “Real” three-dimensional presentation, only visible with 3D glasses (red and cyan glasses).

## Competing interests

The authors declare that they have no competing interests.

## Authors’ contributions

BMS, NL and MK developed the study idea and performed data collection and data analysis. DW and HPM developed the study software and performed data collection. TB performed the statistical analysis. MWB performed data analysis and drafted the manuscript. LF developed the study idea, performed data analysis and drafted the manuscript. All authors read and approved the final manuscript.

## Pre-publication history

The pre-publication history for this paper can be accessed here:

http://www.biomedcentral.com/1472-6920/13/131/prepub
